# Increased Subjective Distaste and Altered Insula Activity to Umami Tastant in Patients with Bulimia Nervosa

**DOI:** 10.3389/fpsyt.2017.00172

**Published:** 2017-09-25

**Authors:** Rikukage Setsu, Yoshiyuki Hirano, Miki Tokunaga, Toru Takahashi, Noriko Numata, Koji Matsumoto, Yoshitada Masuda, Daisuke Matsuzawa, Masaomi Iyo, Eiji Shimizu, Michiko Nakazato

**Affiliations:** ^1^Department of Cognitive Behavioral Physiology, Graduate School of Medicine, Chiba University, Chiba, Japan; ^2^Research Center for Child Mental Development, Chiba University, Chiba, Japan; ^3^School of Nutrition and Dietetics, Kanagawa University of Human Services, Yokosuka, Japan; ^4^Department of Nutrition and Health Sciences, Fukuoka Women’s University, Fukuoka, Japan; ^5^Department of Radiology, Chiba University Hospital, Chiba, Japan; ^6^Department of Psychiatry, Graduate School of Medicine, Chiba University, Chiba, Japan

**Keywords:** eating disorders, bulimia nervosa, umami, glutamate, functional magnetic resonance imaging, neuroimaging, gustation, insula

## Abstract

The aim of this study was to examine differences in brain neural activation in response to monosodium glutamate (MSG), the representative component of umami, between patients with bulimia nervosa (BN) and healthy women (HW) controls. We analyzed brain activity after ingestion of an MSG solution using functional magnetic resonance imaging (fMRI) in a group of women with BN (*n* = 18) and a group of HW participants (*n* = 18). Both groups also provided a subjective assessment of the MSG solution *via* a numerical rating scale. The BN group subjectively rated the MSG solution lower in pleasantness and liking than the control group, although no difference in subjective intensity was noted. The fMRI results demonstrated greater activation of the right insula in the BN group versus the control group. Compared with the HW controls, the BN patients demonstrated both altered taste perception-related brain activity and more negative hedonic scores in response to MSG stimuli. Different hedonic evaluation, expressed as the relative low pleasing taste of umami tastant and associated with altered insula function, may explain disturbed eating behaviors, including the imbalance in food choices, in BN patients.

## Introduction

Bulimia nervosa (BN) is a mental illness characterized by repetitive overeating and purging behaviors to prevent weight gain, and is commonly seen in young women. Patients with BN experience significant psychological, physical, and social suffering related to their ongoing eating disorder (ED), including BN-specific psychopathology. Behavioral abnormalities driven by craving for foods are a prominent feature of BN, and neuroimaging studies using food-related tasks are thus an important approach for elucidating the correlations between these behaviors and their underlying neural substrates (1, 2).

In clinical settings, BN patients describe eating behaviors including anhedonia surrounding mild flavors, pathological consumption of highly palatable foods (e.g., high sugar/fat foods), and diminished feelings of emotional and/or physical satiety (3). Several observational and investigative studies have clarified some skewed preferences in BN-related eating habits. For example, overeating and purging in BN patients are associated with consuming more sucrose and fat, and less protein (4, 5). Also, breads and cereals are the primary carbohydrate sources for healthy eaters, whereas fruits and vegetables are the main carbohydrate sources for BN patients (6). Several studies have indicated that food selection in BN or in postpubertal youth with bulimic symptoms is heavily biased toward increased sugar/fat caloric intake at the expense of protein calories (7, 8). Another study also found high levels of consumption of artificial sweeteners, which provide few calories but excessive sweetness, in BN patients (9). Furthermore, BN patients were unable to enjoy the taste and smell of foods, or to feel distress when they overate (10). These results suggest that BN patients might have some dysfunction in the perception and evaluation of tastes that affects a wide range of foods.

In addition to sweetness, the five basic tastes include saltiness, sourness, bitterness, and umami (11). Umami was discovered in 1908 by isolating monosodium glutamate (MSG) from kelp (12) and is a preferred taste (13). Facial expression studies on neonatal human infant responses to stimulation with different tastes noted that umami-tasting stimuli induced eager sucking and licking movements, as well as triggering facial expressions very similar to those induced by sweet taste (14). MSG, thought to be indicative of the protein content of food (15), is widely used as a flavor-enhancing seasoning that also increases food intake (16). In fact, MSG has several physiological actions. In rats, MSG has obesity-controlling effects (17), and unlike glucose, it does not induce the expression of Fos-related protein in the nucleus accumbens (18). It also induced vagally mediated brain activity when administered intragastrically in rats (19), and promoted gastric motility when added to protein-rich meals in human (20). Behaviorally, the addition of MSG to low-calorie meals enhanced appetite and postprandial satisfaction (21), and when added to essential foods MSG improved balanced food selection in older adults and patients with diabetes (22). Thus, umami is a taste with a certain level of preferentiality, and MSG can potently influence human physiology and nutrition.

Of the many functional magnetic resonance imaging (fMRI) studies of BN patients, those using taste stimuli are considered particularly useful because they directly examine dysfunction in the perception and evaluation of food as a reward (23). To date, fMRI studies of BN patients using taste or flavor stimuli have found abnormal responses to sweet and high-fat cream in specific regions of the brain including the insula, anterior cingulate cortex, orbitofrontal cortex, and ventral striatum (24–28). Although differences across the presented tasks limit comparisons of these studies, Kaye et al. have proposed that patients with EDs have a disorder in the ventral neural pathway formed by the insula, orbitofrontal cortex, amygdala, anterior cingulate cortex, and related regions of the brain, leading to abnormalities in food-related reward evaluation (29). However, those studies were limited by using only highly pleasant tastes, sweetness, or fats.

On another front, it is assumed that the neurophysiology of disgusting stimuli processing may also be important for elucidating the pathogenesis of aberrant eating behaviors in EDs (30, 31). In fact, feelings of disgust influence the modulation of food intake in human (32). Furthermore, BN patients or women with abnormal eating attitudes showed altered disgust sensitivity to food-related stimuli (33–35). More recently, Monteleone et al. investigated the brain response to bitter taste of quinine concomitantly with sweet taste in BN patients (36). Their study focused on the processing of universally aversive taste stimulus, which showed decreased brain response in the amygdala and insula to the bitter taste in BN patients.

Monosodium glutamate has higher hedonic impact than water, but lower impact than sucrose (37). So, it is reasonable to consider that umami does not present high pleasantness on equal terms to sweetness, but it does offer a certain level of pleasantness, contrary to the bitterness that is widely considered as disgusting. In the present study, for the first time, we aimed to investigate the representations of umami in brain of BN patients compared with healthy women (HW). We hypothesized that BN patients show alterations of brain responses to taste based not only on sweetness and fat (obviously pleasant tastes) or bitterness (obviously aversive taste), but also umami (moderately pleasant taste). We thus investigated the brain responses to MSG stimuli in BN patients to determine whether these patients have lost the ability to properly perceive and evaluate umami tastant or its rewarding/satisfying feelings.

## Materials and Methods

### Participants

We recruited 41 women in total, 21 patients with BN and 20 HW participants. Three patients and 2 HW were excluded due to major head motion, greater than 1.0 mm translation or 2.0° rotation, after preprocessing of fMRI, and thus we finally examined a total of 36 women, 18 with BN and 18 healthy participants. BN patients were recruited by clinical referrals from Chiba University Hospital, as well as other local psychiatric hospitals and clinics. Patients were assessed by senior psychiatrists at Chiba University Hospital using the Structured Clinical Interview for DSM-IV Axis I Disorders, Research Version, Patient Edition (SCID-I/P) (38). Most of the patients were to undergo cognitive-behavioral therapy at the Department of Psychiatry of Chiba University Hospital after this study. All patients met the current DSM-IV criteria for BN. Four patients reported comorbid depression, while two patients had used selective serotonin reuptake inhibitors (sertraline and fluvoxamine, respectively). No patients had met criteria for drug or alcohol abuse or dependence, mental retardation, current high risk of suicide, or psychotic symptoms. HW participants matched for age and gender were recruited through local advertisements. All HW participants were without a history of EDs or any psychiatric, serious medical or neurological illness, and they were not on medication. All participants were female native Japanese speakers, between age 18 and 36 (mean = 26.10, SD = 5.68 years). The Research Ethics Committee of Chiba University Hospital approved all procedures, and written informed consent was obtained from all participants prior to scanning. All experiments were performed in accordance with the Helsinki Declaration.

### fMRI Task Paradigm

The magnetic resonance imaging study was done at 16:00 o’clock, 4 h after ingestion of a meal. The trial was registered as UMIN ID: 0000010220. For this study, an aqueous solution of 0.05 M MSG plus 0.102 M (0.6%) sodium chloride was used as stimulus. Salt supplementation was used because the taste intensity experienced with MSG is related to the NaCl concentration (39). Moreover, NaCl is often present in foods containing MSG, so supplemented MSG actually represents a more natural taste stimulus (39). In a preliminary study, we noted that the addition of 0.102 M sodium chloride to MSG solution adequately increased the subjective intensity of taste (3). The concentration of MSG was determined by reference to our and others’ preliminary studies (3, 22, 40). Distilled water was used to control for activation related to receiving and swallowing a liquid. The rinsing effect also prevented smearing of the stimulus and enhanced its perception. The MSG solution and water were delivered into the participant’s mouth *via* separate polyethylene tubes at room temperature (24°C). The tubes were connected to a mouthpiece held between the lips, and the tips of the tubes were placed in the oral cavity to enable delivering solutions to the anterior domain of the tongue. Solutions were delivered with syringe pumps manually at the time indicated by computer and a trained technician who prepared the start according to a signal 1 s before delivery. The tubes were filled with the respective solutions by syringe prior to the scanning sessions.

**Figure 1:**
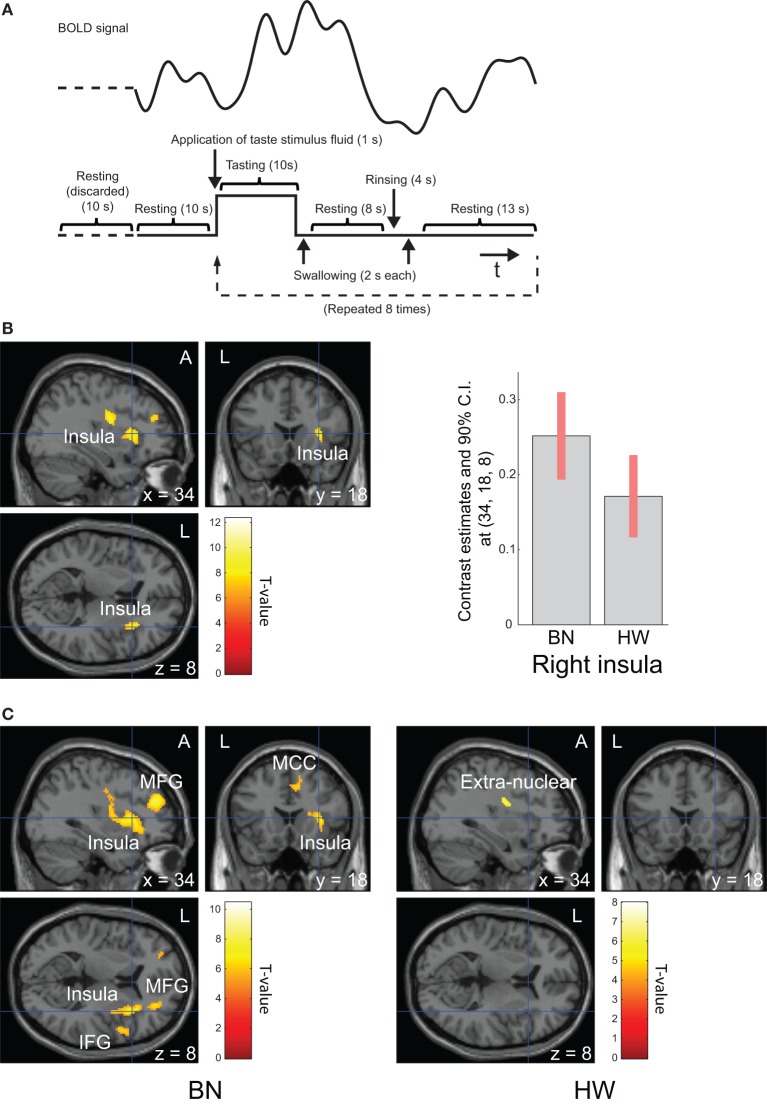
Task design and brain regions activated during tasting monosodium glutamate (MSG) solution. Functional magnetic resonance imaging task design, timeline of events, a representative timecourse of observed blood-oxygen-level dependent (BOLD) responses **(A)**. Brain regions activated during tasting the MSG solution for all participants (**B**, left) and patients with BN and HW **(C)**. BOLD signal responses to MSG stimuli [**(B)**, right]. Statistical maps were thresholded at *P* < 0.001 with familywise error (FWE) correction at peak level **(B)** or *P* < 0.05 with FWE correction at peak level **(C)**. Then, both were thresholded at *q* < 0.05 with false discovery rate correction at cluster level. During tasting solution, BOLD signal response was shown in the right anterior insula for all participants. The bar graph [**(B)**, right] represents relative activations in the right insula (*x, y, z* = 34, 18, 8) in response to MSG stimuli between the two groups. The error bar represents 90% of CI. A, anterior; BN, patients with bulimia nervosa; CI, confidence interval; HW, healthy women participants; L, left.

Before scanning, the participants were instructed as follows: “(i) In this examination, you will be administered a taste solution *via* a mouthpiece repeatedly. (ii) In line with the administration of the solution, roll the solution around on the tongue and continue tasting. (iii) According to the instructions on the screen, swallow the solution.” Each trial was defined by the following sequence: application of taste stimulus fluid (1 s), tasting (10 s), swallowing (2 s), resting (8 s), rinsing (4 s), swallowing (2 s), and resting (13 s, Figure 1A). The taste stimulus consisted of 0.7 ml volume of the MSG solution given as a bolus. Participants were instructed to swallow the fluid before the first “resting” period. The instructions were provided *via* a screen hung on the top of the bore that the participants viewed using an angled mirror mounted above the head coil. After “tasting,” “swallowing,” and the first “resting,” the participant was delivered 0.7 ml of distilled water. Participants were instructed to swallow the water before the second “resting” period in the same way. Trials were repeated regularly eight times after looking at a fixation cross for 20 s. Total scan time was 340 s.

### fMRI Acquisition and Image Processing

At Chiba University, all participants underwent MR imaging using a scanner equipped with a 32-channel phased-array head coil (Discovery MR750 3.0 T, General Electric Healthcare, Waukesha, WI, USA). Images were collected by gradient-recalled echo-planar imaging sequence (echo time, 30 ms; repetition time, 2,000 ms; number of slices, 36; flip angle, 76°; acquisition matrix, 64 × 64; slice thickness, 3.7 mm; field of view, 24.0 cm; bandwidth, 111.11 kHz). fMRI images were analyzed using SPM8 software (Wellcome Institute of Neurology, University College London, London, UK, http://www.fil.ion.ucl.ac.uk/spm) running under MATLAB^®^2013a (The MathWorks, Inc., Natick, MA, USA). Data from the first five volumes were discarded to avoid transient magnetization. Other volumes were realigned to the first one of each scanning session to correct for subject motion, and were spatially normalized into the standard space defined by the Montreal Neurological Institute template. Then, the images were smoothed with an 8-mm isotropic Gaussian kernel.

### fMRI Analysis

First level contrast images for each participant were generated on a voxel-by-voxel basis using a general linear model with the “tasting” period (10 s in each trial) as task conditions and the remaining period (“water rinsing” period and waiting phase before and after water rinsing that comprise the total 30 s) as baseline conditions. The design matrix employed a box-car function convolved with the canonical hemodynamic response function, with time and dispersion derivatives. Realignment parameters were included as a nuisance covariate to control for head motion. Next, in order to confirm task-related activation in the whole brain, one-sample *t*-tests were performed for all participants’ data [*P* < 0.001 with familywise error (FWE) correction at peak level and *q* < 0.05 with false discovery rate (FDR) correction at cluster level]. Such tests were also conducted for each group’s data (*P* < 0.05, with FWE correction at peak level and *q* < 0.05 with FDR correction at cluster level). Thirdly, in order to detect a group difference in the response to MSG stimuli between the BN and control groups, we performed two-sample *t*-tests on the effects of tasting the MSG solution (small volume corrections, *P* < 0.05, FWE corrected at the voxel level) after *a priori* whole-brain analysis thresholded at *P* < 0.001, uncorrected for multiple comparisons and more than 54, which was computed as the number of expected voxels per cluster. It is known that populations affected by depressive or anxiety symptoms are characterized by a higher disgust sensitivity threshold (41), which might affect food intake (42). In addition, there is evidence that anxiety itself can affect food intake (42). So, we additionally investigated group differences with depression and anxiety scores as nuisance covariates, respectively. Regions-of-interest in taste processing, which are the left and right insula and anterior cingulate cortex, were defined using the automated anatomical labeling atlas in the PickAtlas (43) for small volume correction.

### Questionnaire-Based Measurements

Current symptoms were assessed by the Eating Disorders Examination Questionnaire (EDE-Q) (44), Bulimic Investigatory Test, Edinburgh (BITE) (45), Patient Health Questionnaire (PHQ-9) (46), and Generalized Anxiety Disorder scale (GAD-7) (47). EDE-Q is a well-known self-report questionnaire that assesses an ED-related symptom profile. EDE-Q includes four subscale scores—restricting (EDE-Qr), eating concern (EDE-Qe), shape concern (EDE-Qs), and weight concern (EDE-Qw). Each subscale score is the mean score of the items forming that subscale, while the global score (EDE-Qg) is the mean of the four subscale scores. The BITE is a 33-item self-report questionnaire designed to assess symptoms of bulimia or binge eating. BITE is composed of two subscales: the symptom assessment scale (BITE-sas) and the severity scale (BITE-ss). The former measures the degree of symptoms present, and the latter provides an index of the severity of binging and purging behaviors as defined by their frequency. PHQ-9 is an easy-to-use screening tool for assessing current depressive symptoms, with a total score >10 (on a 0–27 scale) indicating clinically significant depression. GAD-7 is a simple assessment tool to measure a participant’s current anxiety, based on a total score range of 0–21 (higher scores represent greater anxiety). Immediately after scanning, participants were asked to complete a questionnaire including the existence of the feeling of umami, saltiness, and any other taste such as sweetness, sourness, and bitterness in the solution as well as subjective ratings for pleasantness, preference, and intensity of taste for the solution received in the scanner, with numerical ratings on a scale from 5 (extremely pleasant) to −5 (extremely unpleasant), including 0 (neutral), from 5 (extremely like) to −5 (extremely dislike), including zero (neutral), and 10 (extremely intense) to 0 (tasteless), respectively.

### Statistical Analyses

The non-parametric correlations among the subjective ratings of taste, each of the scales forthe clinical measures (EDE-Qg, EDE-Qr, EDE-Qe, EDE-Qw, EDE-Qs, BITE-ss, BITE-sas, PHQ-9, and GAD-7), and the eigenvariate of the peak voxel defined in the primary gustatory regions (48, 49) activated with the MSG solution in all participants were calculated using SPSS version 21 (IBM Corp., Armonk, NY, USA).

## Results

### Characteristics of Participants

Demographic and clinical characteristics of female BN patients and HW participants are summarized in Table 1. The two groups did not differ in terms of age, period of education, body mass index, and handedness. On the other hand, BN patients exhibited higher symptoms and severities of ED, depression, and anxiety [EDE-Qg, BN (mean): 4.29, HW (mean): 1.31, *P* < 0.001; BITE-ss, BN (mean): 13.17, HW (mean): 1.44, *P* < 0.001; BITE-sas, BN (mean): 24.44, HW (mean): 6.56, *P* < 0.001; PHQ-9, BN (mean): 12.83, HW (mean): 4.56, *P* < 0.001; GAD-7, BN (mean): 8.39, HW (mean): 4.78, *P* = 0.022]. Mann–Whitney *U* tests revealed that the BN group differed significantly from the control group in all of the dimensional assessments. BN patients reported not only symptoms of BN, but also higher depressive and anxiety symptoms, compared with HW participants.

**Table 1:** Demographic and clinical data of patients with bulimia nervosa (BN) and healthy women (HW) participants.

	BN			HW			*P*-value
	Mean	SD	Range	Mean	SD	Range	
Number of participants (*n*)	18			18			
Age (years)	25	5.63	(18.6–36.3)	27.09	5.69	(20.7–36.4)	0.285^a^
Education (years)	13.89	1.94	(11–18)	14.56	1.04	(12–16)	0.211^a^
Duration of illness (years)	3.25	3.41	(0.6–6.5)	–	–	–	–
BMI (kg/m^2^)	20.21	1.62	(18.2–23.7)	20.51	1.44	(18.8–22.8)	0.55^a^
Handedness (L/R; *n*)	1/17			1/17			
EDE-Qg	4.29	1.11		1.31	0.71		<0.001^b^
EDE-Qr	4.12	1.56		0.6	0.62		<0.001^b^
EDE-Qe	3.94	1.23		0.67	0.57		<0.001^b^
EDE-Qw	4.5	1.13		1.74	1.16		<0.001^b^
EDE-Qs	4.58	1.23		2.17	1.21		<0.001^b^
BITE-ss	13.17	4.94		1.44	0.98		<0.001^b^
BITE-sas	24.44	3.05		6.56	4.2		<0.001^b^
PHQ-9	12.83	5.37		4.56	3.2		<0.001^b^
GAD-7	8.39	5.07		4.78	3.26		0.022^b^
Comorbidities (*n*)							
Major depressive disorder	4			–			
Medication (*n*)							
SSRIs	2			–			

*BMI, body mass index; BITE, Bulimic Investigatory Test, Edinburgh; BITE-sas, the symptom assessment scale of BITE; BITE-ss, the severity scale of BITE; EDE-Q, Eating Disorders Examination Questionnaire; EDE-Qe, eating concern; EDE-Qg, global score, the average of the four subscale scores; EDE-Qr, restricting; EDE-Qs, shape concern; EDE-Qw, weight concern; GAD-7, Generalized Anxiety Disorder scale; PHQ-9, Patient Health Questionnaire; SSRIs, selective serotonin reuptake inhibitors*.

*^a^Two-sample t-tests*.

*^b^Mann–Whitney U tests*.

### Subjective Evaluations of the Taste Solution

All participants replied that they felt umami and saltiness to the taste stimulus (0.05 M MSG in 0.102 M NaCl). No participants discerned any tastes other than umami and saltiness. Subjective ratings for the taste solution are summarized in Table 2. Significant differences in the pleasantness [−5 to 5 scale, BN (mean): −1.44, HW (mean): 0.61, *P* = 0.006] and preference [−5 to 5 scale, BN (mean): −0.67, HW (mean): 1.11, *P* = 0.047] ratings were observed between BN and HW. BN patients experienced relatively low pleasantness and liking with the MSG solution. On the other hand, there was no significant difference in the intensity of taste ratings between the BN and control groups [0–10 scale, BN (mean): 6.16, HW (mean): 5.33, *P* = 0.152].

**Table 2:** Numerical rating scale for pleasantness, preference, and intensity with taste solution.

	Bulimia nervosa		Healthy women		*P*-value
	Mean	SD	Mean	SD	
Pleasantness (between −5 and 5)	−1.44	2.25	0.61	1.79	0.006^a^
Preference (between −5 and 5)	−0.67	2.74	1.11	2.17	0.047^a^
Intensity (between 0 and 10)	6.16	1.82	5.33	1.41	0.152^a^

*^a^Mann–Whitney U tests*.

### Main Effect of Neural Activation during Tasting MSG Solution

Whole-brain analysis in all participants revealed robust activation in the bilateral precentral/postcentral gyrus, bilateral superior frontal gyrus, right anterior insula, left cerebellum, and right middle frontal gyrus while tasting MSG (*P* < 0.001 FWE correction at peak level and *q* < 0.05 with FDR correction at cluster level, Table 3 and Figure 1B). In each group analysis, bilateral precentral/postcentral gyrus, right inferior frontal gyrus, insula, bilateral supplementary motor area, middle frontal gyrus, left superior frontal gyrus, and left cerebellum were activated in BN patients, and bilateral precentral/postcentral gyrus was activated in HW participants (*P* < 0.05 FWE correction at peak level and *q* < 0.05 with FDR correction at cluster level, Table 3; Figure 1C).

**Table 3:** Brain regions activated during tasting monosodium glutamate solution.

Region	Hemisphere	MNI coordinates			*Z*-score	Voxels	*P*^FWE^-value
		*x*	*y*	*z*			
**All participants**							

Precentral/postcentral gyrus	L	−60	−12	30	7.61	998	<0.001
	L	−44	−18	38	6.54		<0.001
	L	−62	6	22	5.57		<0.001

Precentral/postcentral gyrus	R	56	−6	38	7.00	1,891	<0.001
	R	54	−6	30	7.00		<0.001
	R	64	4	22	6.86		<0.001

Superior frontal gyrus	L	−8	−6	60	6.53	487	<0.001
	R	8	0	60	6.45		<0.001

Insula	R	34	18	8	6.25	155	<0.001
	R	38	10	2	5.66		<0.001

Cerebellum	L	−36	−62	−34	5.94	126	<0.001
	L	−12	−66	−28	5.70		<0.001
	L	−20	−68	−32	5.54		<0.001

Middle frontal gyrus	R	32	42	24	5.93	55	<0.001

**BN**							

Precentral/postcentral gyrus	L	−58	−14	30	6.94	1,085	<0.001
	L	−44	−18	40	6.13		<0.001
	L	−64	−8	18	5.56		<0.001

Precentral/postcentral gyrus/inferior frontal gyrus/insula	R	50	−8	30	6.22	3,454	<0.001
	R	32	42	26	6.12		<0.001
	R	56	−6	40	5.96		<0.001

Supplementary motor area/middle frontal gyrus	R	10	0	62	5.83	956	<0.001
	L	−4	10	50	5.52		0.001
	L	−2	−2	60	5.36		0.001

Superior frontal gyrus/middle frontal gyrus	L	−30	46	20	5.48	333	0.001
	L	−26	48	6	4.74		0.018

Cerebellum	L	−36	−62	−34	5.15	252	0.003
	L	−12	−64	−28	4.86		0.011

**HW**							

Precentral/postcentral gyrus	L	−60	−12	30	5.94	319	<0.001
Precentral/postcentral gyrus	R	58	−4	38	5.51	908	0.001
	R	62	4	20	5.44		0.001
	R	30	−4	22	5.40		0.001

*Activated brain regions are defined by thresholding at *P* < 0.001 (for all participants) or *P* < 0.05 (for each group) with FWE correction at peak level, and *q* < 0.05 with false discovery rate correction at cluster level*.

*BN, patients with bulimia nervosa; FWE, familywise error; HW, healthy women participants; MNI, Montreal Neurological Institute*.

**Figure 2:**
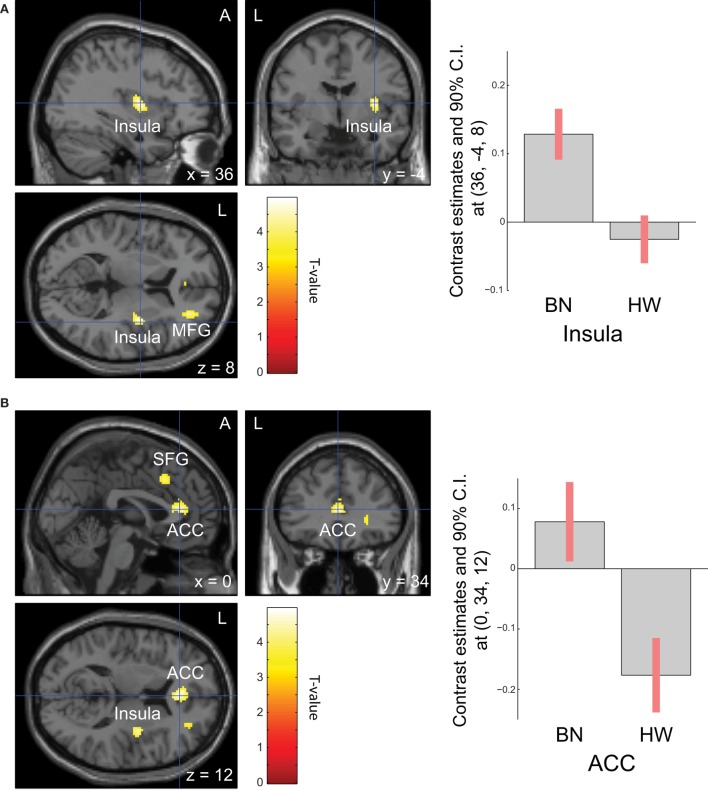
Group differences of blood-oxygen-level dependent signal responses to monosodium glutamate (MSG) stimuli between patients with BN and HW. The BN group showed higher activation in the right middle insula, bilateral superior frontal gyrus, anterior cingulate cortex, and right middle frontal gyrus compared with the control group at the initial whole-brain exploration threshold at *P* < 0.001 uncorrected for multiple comparisons and more than 54 voxels, which was computed as the number of expected voxels per cluster. No brain regions showed smaller activation in the BN group compared with the control group. Statistical maps are shown in sections of regions-of-interest of right insula [**(A)**, left] and anterior cingulate [**(B)**, left]. The bar graphs represent contrast estimates in the right middle insula [*x, y, z* = 36, −4, 8, **(A)**, right] and the anterior cingulate cortex [*x, y, z* = 0, 34, 12, **(B)**, right] in response to MSG stimuli in the two groups. The error bar represents 90% of CI. A, anterior; ACC, anterior cingulate cortex; BN, patients with bulimia nervosa; CI, confidence interval; HW, healthy women participants; L, left; MFG, middle frontal gyrus; SFG, superior frontal gyrus.

When analyzing the group differences in response to MSG stimuli, the BN group showed higher activation in the right middle insula, bilateral superior frontal gyrus, anterior cingulate cortex, and right middle frontal gyrus compared with the control group (*P* < 0.001 uncorrected for multiple comparisons), of which the right insula and anterior cingulate cortex survived after small volume correction (*P* < 0.05, Table 4 and Figure 2). Brain regions with greater activity in the BN group compared with the control group, and bar graphs of the contrast estimates at the right middle insula (Figure 2A) and dorsal anterior cingulate cortex (Figure 2B) were shown. No brain region showed greater activity in response to MSG stimuli in the control group than in the BN group. In additional analysis with the covariates of depression and anxiety, significant neural response difference in the right insula between the BN group and HW group survived after controlled for depression and anxiety, respectively (Tables S1 and S2 in Supplementary Material).

**Table 4:** Group differences of BOLD signal responses to monosodium glutamate stimuli.

Region	Hemisphere	MNI coordinates			*Z*-score	Voxels	*P*^SVC^-value
		*x*	*y*	*z*			
**BN > HW**							
Insula	R	36	−4	8	4.27	47	0.002
Anterior cingulate cortex	0	34	12	4.06	150	0.006
**HW > BN**							
None							

*Small volume corrections (*P* < 0.05, familywise error corrected at voxel level) were applied after *a priori* whole-brain analysis thresholded at *P* < 0.001 uncorrected for multiple comparisons and more than 54 voxels, which was computed as the number of expected voxels per cluster*.

*BN, patients with bulimia nervosa; BOLD, blood-oxygen-level dependent; HW, healthy women participants; MNI, Montreal Neurological Institute; SVC, small volume correction*.

### Correlation among Brain Activation to MSG Stimuli and Subjective Ratings for the Taste Solution, and Clinical Measures

**Figure 3:**
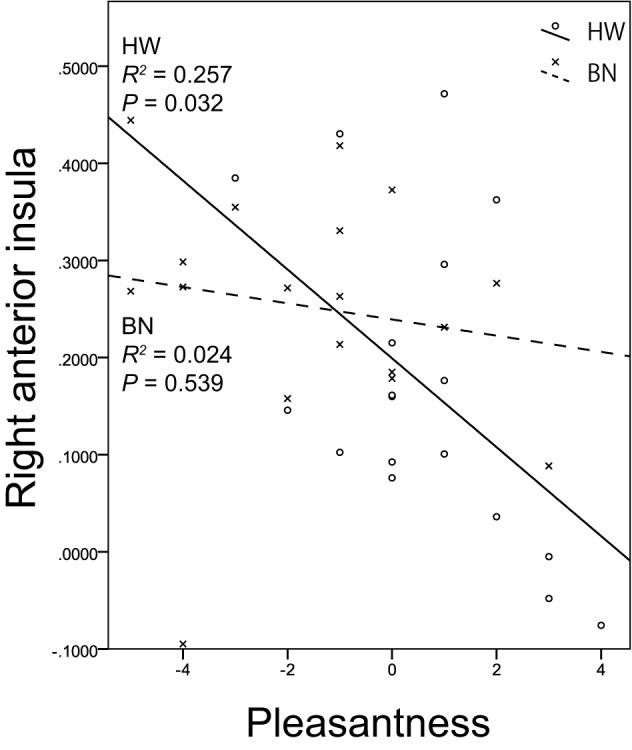
The relationship between brain activation in the right anterior insula related to umami taste and subjective hedonic ratings for the taste solution. The *x*-axis shows the subjective ratings for the taste solution by the participants, and the *y*-axis shows contrast estimates in the right anterior insula (*x, y, z* = 34, 18, 8). Scatter plots show the significant negative correlation between brain activation related to monosodium glutamate stimuli in the right anterior insula and pleasantness in HW participants indicated by the solid lines. No correlations were found in BN patients indicated by the dashed lines. BN, patients with bulimia nervosa; HW, healthy women participants.

Non-parametric tests revealed a correlation between brain activity in the right anterior insula (*x, y, z* = 34, 18, 8) and the subjective rating of pleasantness for the taste solution in HW participants. Figure 3 shows the associations between the brain activities in the right anterior insula caused by MSG stimuli and pleasantness in both groups. A certain degree of brain activity was observed in the right anterior insula of the BN group regardless of the score of pleasantness for the MSG solution. In contrast, right anterior insular brain activity in the HW group decreased with increasing pleasantness. No other correlations between brain activity and subjective ratings were found in either the BN or HW group. Furthermore, no correlations were found between brain activity and each of the clinical measures (EDE-Qg, EDE-Qr, EDE-Qe, EDE-Qw, EDE-Qs, BITE-ss, BITE-sas, PHQ-9, and GAD-7) in either group. Also, no correlations were found between subjective ratings for the taste solution and each of the clinical measures in either group.

## Discussion

To the best of our knowledge, this is the first study to investigate brain activation in response to tasting umami tastant in BN patients. We expected that the brain regions related to food-reward processing, such as the insula, orbitofrontal cortex, anterior cingulate cortex, and ventral striatum (50, 51), would show aberrant activation in BN patients, based on previous studies showing altered activation in these regions in BN with regard to sweet taste (24–27) or high-fat cream (28). Our data showed higher activation in the insula and anterior cingulate cortex, but no difference in the orbitofrontal cortex and ventral striatum, in BN patients compared with HW participants.

Monosodium glutamate tasting-related activations were strongly seen in the right insula in all participants. This is consistent with previous fMRI studies showing umami-related brain activation in healthy individuals in the insula (40, 52, 53). Thus, the MSG tasting task we employed appeared appropriate as a taste-presenting task.

The most important result in our study is that BN patients showed higher activation in the right insula compared with HW participants. The insula consolidates the sensations of taste, temperature, viscosity, and texture (54), and it is interconnected with the orbitofrontal cortex, anterior cingulate cortex, amygdala, and other regions of the brain responsible for evaluating the comfort and emotional relevance of taste stimuli in relation to previous experiences (50, 51). In addition, it has multimodal roles for the integration of affective, cognitive, and physiological information (55). Thus, the insula is a pivotal structure in the corticolimbic circuits involved in appetite regulation, integrating both internal and external information linked to reward processing, whereas the homeostatic aspects of the regulation of food intake are mediated by the hypothalamus (56). The altered activation in the middle insula in BN in the present study might be interpreted as the altered representation of MSG taste in the primary taste cortex of the human brain. It has been regarded that the primary taste cortex in the human brain is composed of the anterior insula and adjoining frontal operculum (48, 49). In fact, these areas were activated in response to each prototypical taste stimulus, sweet, and salty (57), bitter and sour (53), and umami (40). However, recent findings from neuroimaging studies suggest that gustatory information may be represented in the more posterior parts of the human insular region (58–60). In fact, multiple studies demonstrated sufficient activation to taste stimuli in the middle or posterior insula, as well as in the anterior insula, in humans (57, 61–63). Considering these data, the location of the primary taste cortex in the human insular cortex is less precisely defined. Thus, our data might indicate altered representation of MSG taste in the primary taste cortex in BN patients. The primary taste cortex acts as the entry point into the corticolimbic circuits involved in appetite regulation that represent the quality and intensity of the taste of food (64). In our study, the subjective ratings of intensity to the MSG solution were not significantly different between the two groups. Thus, our finding in the insula might be interpreted as the representation of altered perception and evaluation to quality of the MSG solution in BN.

Although the brain activity in the right insula in the HW group increased with decreasing subjective pleasantness for the MSG stimuli, that in the BN group was not affected. Interestingly, several studies reported that brain activation to tastes in the right insula decreased as unpleasantness of taste decreased in healthy subjects (65, 66). Our result is consistent with those findings. On the other hand, BN patients showed deregulated insula activation regardless of the scores of pleasantness of the MSG solution. These results also reinforce our hypothesis of an inability in BN patients to evaluate umami taste properly.

In our results, BN patients showed higher activation in the dorsal anterior cingulate cortex, whereas the orbitofrontal cortex and pregenual anterior cingulate cortex, regions most often reported in co-activation with the orbitofrontal cortex in taste-related studies (67), were not significantly activated in the two groups. The dorsal anterior cingulate cortex is known to reflect the unpleasantness of taste stimuli (68, 69). Higher activation in the dorsal anterior cingulate cortex to MSG stimuli in BN patients compared with HW participants might reflect the perceived relatively low pleasantness or liking of the taste in BN patients.

As previously mentioned, some fMRI studies have investigated the brain responses to taste stimuli in BN with regard to food-reward processing errors in BN. Patients with current BN showed reduced anticipatory responses in the insula to a (sweet) chocolate milkshake (24), and in the insula and striatum to the sweet taste of sucrose (26). Individuals who had recovered from BN showed increased responses in the insula to sucrose (27), and in the striatum to high-fat flavor (whipping cream) (28). These studies showed reduced anticipatory and exaggerated consummatory responses to taste stimuli with hedonic impact, suggesting that this pattern of responding to food-reward might account for deficits in reward evaluating before eating, and excessive reward evaluating in food consumption, which might support the disturbed eating behavior in BN. The enhanced insula activity in the consummatory phase found in our study is consistent with the results of these previous studies, although there are conflicting results among them. Frank et al. reported that participants with recovered BN showed decreased activity in the right anterior cingulate cortex and left cuneus when tasting glucose (25). Bohon and Stice also reported reduced activity in the right insula of patients with current BN related to consummatory, and not just anticipatory, responses to chocolate milkshake (24). To establish a consistent theory on the taste response pattern of BN patients with regard to food-reward processing errors, more data must be accumulated for the future studies.

From a different perspective, it is notable that the insula might work as the key neural structure responsible for disgust processing (70, 71). Vicario et al. suggests that the insula might be the hub structure linking each domain of disgust, not just food-related disgust, in consort with other interconnected brain regions such as the orbitofrontal cortex, striatum, and anterior cingulate cortex (41). Although little is known concerning BN, the involvement of insula dysfunction to altered disgust processing is well-known in AN, and in people with hyperphagia such as obesity (41). Furthermore, as previously mentioned, a recent fMRI study revealed altered insula response to the highly aversive stimulation that is bitterness with quinine in BN patients (36). Since the possibility exists that BN patients might perceive the MSG solution as an aversive stimulus, our results which showed higher activation in the right insula and more negative hedonic scores in response to MSG stimuli might indicate altered processing of disgust not only to highly aversive taste stimuli but also to a wide range of taste stimuli in the current BN patients.

Sweetness, a widely preferred taste, has intense hedonic impact (72), and it may lead to binging or overconsumption (73). On the other hand, MSG also has a greater hedonic impact than water, but a lower impact than sucrose (37). The present study indicates that BN patients might have impaired function in evaluating not only highly pleasant tastes but also tastes with mild pleasantness such as umami, with the possibility that they tend to perceive a wide range of tastes as disgust. Thus, our study suggests the importance of not only controlling excessive palatable foods, but also supporting restoration of appreciation for moderate taste in the therapeutic practice for patients with BN.

Several limitations of the present study should be noted. First, because of a lack of accurate and objective examination methods, we were unable to investigate the actual eating habits and preferences of the participants in this study. Also standardized meals prior to the scanning and assessment of subjective ratings on the distilled water and satiety level during scanning would allow for a more accurate analysis of the brain responses to umami tastant. Second, this fMRI study focused only on umami tastant; sweetness and other taste stimuli were not tested. If sweetness and the other tastes were compared simultaneously in the same participants, the significance and specificity of brain responses to umami tastant would be clearer. Furthermore, we did not measure disgust sensitivity and disgust propensity in our participants. Considering the involvement of their factors in insula activation to aversive stimuli (74), assessment of disgust sensitivity and disgust propensity might help us to attain more reasoned interpretation of our results. In addition, the participants in the study were all Japanese women, who are obviously familiar with Japanese diet and dietary culture, which is deeply involved in applying umami flavoring to various dishes. There are clear inter-individual differences in sensitivity to MSG, and in familiarity to the umami taste, in European cultures (75). Our design limited the generalizability of our conclusions, especially in people of other cultures. Further, we did not exclude participants with depression and those taking medication, although they comprised a small number. Although significant neural response difference in the right insula between the BN group and HW groups survived after control for depression and anxiety (Tables S1 and S2 in Supplementary Material, respectively), our results could have been influenced by these factors. Another limitation relates to our task not excluding the effects of anticipation for taste stimuli on neural responses. Future studies that investigate independently both consummatory and anticipatory phases will help understand neural substrates in patients with BN more clearly.

In conclusion, this is the first study that investigated brain responses to umami tastant in BN patients. The results demonstrated altered perception and hedonic evaluation to umami tastant in BN patients, which might contribute to imbalance in food choices and disordered feelings of satisfaction and pleasure when eating a well-balanced diet.

## Ethics Statement

The Research Ethics Committee of Chiba University Hospital approved all procedures, and written informed consent was obtained from all participants prior to scanning. All experiments were performed in accordance with the Helsinki Declaration.

## Author Contributions

RS, YH, and MN designed the project and RS, MT, and TT prepared and provided the MSG solution. RS, YH, NN, KM, and YM. performed the experiment and collected the data. YH and RS analyzed the data. RS and YH drafted the manuscript. MN, DM, MI, and ES supervised this study.

## Conflict of Interest Statement

The authors declare that the research was conducted in the absence of any commercial or financial relationships that could be construed as a potential conflict of interest.
