# Supplementary material for: Increased Subjective Distaste and Altered Insula Activity to Umami Tastant in Patients with Bulimia Nervosa
Source: Front Psychiatry. 2017 Sep 25;8:172. doi: 10.3389/fpsyt.2017.00172 (PMC5622337; doi:10.3389/fpsyt.2017.00172)
Supplement: Supplementary file 1 [file table_1.docx]

**Table S1.** Group differences of BOLD signal responses to MSG stimuli controlled for depression

|  | | | | | | | | |
| --- | --- | --- | --- | --- | --- | --- | --- | --- |
|  |  |  |  | MNI coordinates |  |  |  |  |
| Region |  | Hemisphere | x | y | z | Z-score | Voxels | *P*^SVC^ |
| BN > HW |  |  |  |  |  |  |  |  |
| Insula | | R | 40 | 2 | 0 | 3.60 | 61 | 0.027 |
| HW > BN |  |  |  |  |  |  |  |  |
| None |  |  |  |  |  |  |  |  |
|  |  |  |  |  |  |  |  |  |

Small volume corrections (P < 0.05, family-wise error corrected at voxel level) were applied after a priori whole-brain analysis thresholded at P < 0.001 uncorrected for multiple comparisons and more than 53 voxels, which was computed as the number of expected voxels per cluster. BN, patients with bulimia nervosa; BOLD, blood-oxygen-level dependent; HW, healthy women participants; MNI, Montreal Neurological Institute; SVC, small volume correction
